# Homozygous Ser-1 to Pro-1 mutation in parathyroid hormone identified in hypocalcemic patients results in secretion of a biologically inactive pro-hormone

**DOI:** 10.1073/pnas.2208047120

**Published:** 2023-02-16

**Authors:** Patrick Hanna, Ashok Khatri, Shawn Choi, Severine Brabant, Matti L. Gild, Marie L. Piketty, Bruno Francou, Dominique Prié, John T. Potts, Roderick J. Clifton-Bligh, Agnès Linglart, Thomas J. Gardella, Harald Jüppner

**Affiliations:** ^a^Endocrine Unit, Massachusetts General Hospital and Harvard Medical School, Boston, MA 02114; ^b^Université Paris Cité, Institut Necker Enfants Malades, Institut National de la Santé et de la Recherche Médicale U1151, Service des Explorations Fonctionnelles, Hôpital Necker Enfants Malades, Assistance Publique – Hôpitaux de Paris, Paris 75015, France; ^c^Department of Endocrinology Royal North Shore Hospital, St Leonards, NSW 2065, Australia; ^d^Sydney Medical School, Faculty of Medicine and Health, University of Sydney, Camperdown, NSW 2050, Australia; ^e^Cancer Genetics Laboratory, Kolling Institute of Medical Research, St. Leonards, NSW 2064, Australia; ^f^Université Paris-Saclay, Institut National de la Santé et de la Recherche Médicale, Physiologie et Physiopathologie Endocrinienne, Assistance Publique – Hôpitaux de Paris, Department of Molecular Genetics, Bicêtre Paris-Saclay Hospital, Le Kremlin Bicêtre 94270, France; ^g^Assistance Publique – Hôpitaux de Paris, Endocrinology and Diabetology for Children, Bicêtre Paris Saclay Hospital, Le Kremlin Bicêtre 94270, France; ^h^Université Paris Saclay, Institut National de la Santé et de la Recherche Médicale, Physiologie et Physiopathologie Endocrinienne, Bicêtre Paris Saclay Hospital, Le Kremlin Bicêtre 94270, France; ^i^Pediatric Nephrology Unit, Massachusetts General Hospital and Harvard Medical School, Boston, MA 02114

**Keywords:** PTH, calcium, phosphate, hypoparathyroidism, pseudohypoparathyroidism

## Abstract

Like other secreted peptides, nascent parathyroid hormone (PTH) is synthesized with a pre- and a pro-sequence (25 and 6 amino acids, respectively). These precursor segments are sequentially removed in parathyroid cells before packaging into secretory granules. Three patients from two unrelated families who presented during infancy with symptomatic hypocalcemia were found to have a homozygous serine (S) to proline (P) change affecting the first amino acid of the mature PTH. Unexpectedly, biological activity of synthetic [P1]PTH(1-34) was indistinguishable from that of unmodified [S1]PTH(1-34). However, in contrast to conditioned medium from COS-7 cells expressing prepro[S1]PTH(1-84), medium from cells expressing prepro[P1]PTH(1-84) failed to stimulate cAMP production despite similar PTH levels when measured by an intact assay that detects PTH(1-84) and large amino-terminally truncated fragments thereof. Analysis of the secreted, but inactive PTH variant led to the identification of pro[P1]PTH(−6 to +84). Synthetic pro[P1]PTH(−6 to +34) and pro[S1]PTH(−6 to +34) had much less bioactivity than the corresponding PTH(1-34) analogs. Unlike pro[S1]PTH(−6 to +34), pro[P1]PTH(−6 to +34) was resistant to cleavage by furin suggesting that the amino acid variant impairs preproPTH processing. Consistent with this conclusion, plasma of patients with the homozygous P1 mutation had elevated proPTH levels, as determined with an in-house assay specific for pro[P1]PTH(−6 to +84). In fact, a large fraction of PTH detected by the commercial intact assay represented the secreted pro[P1]PTH. In contrast, two commercial biointact assays that use antibodies directed against the first few amino acid residues of PTH(1-84) for capture or detection failed to detect pro[P1]PTH.

## Significance

Three patients with symptomatic hypocalcemia had much elevated plasma levels of PTH when measured by intact PTH immunoassay, but low or undetectable levels in biointact PTH assays, thus supporting the diagnosis of either pseudohypoparathyroidism or hypoparathyroidism. A homozygous nucleotide change was identified in all three cases that leads to replacement of serine (S) at position 1 of mature PTH with proline (P). Surprisingly, synthetic [P1]PTH(1-34) and [S1]PTH(1-34) had indistinguishable bioactivity in vitro. However, cells transfected with plasmids encoding wild-type or mutant preproPTH showed that the P1 mutation leads to secretion of biologically inactive pro[P1]PTH(−6 to +84). Consistent with these cell-based findings, pro[P1]PTH was readily detectable in plasma of our patients when using an immunoassay specific for the mutant pro-hormone.

Secreted, biologically active parathyroid hormone (PTH), which comprises 84 amino acids, is synthesized by the parathyroid glands as preproPTH (1, 2). The hydrophobic pre-sequence of 25 amino acids is cleaved off in the rough endoplasmic reticulum, while the positively charged pro-sequence of 6 amino acids (Lysine-Serine-Valine-Lysine-Lysine-Arginine (KSVKKR)) is removed in the trans-Golgi network (3) by a pro-protein convertase (PC), most likely furin and/or PC-7 (4, 5). This leads to the generation of secreted PTH(1-84), the most important peptide hormone regulator of calcium homeostasis that also regulates urinary phosphate excretion.

PTH binds to the PTH/PTHrP receptor (PTHR1), a G protein-coupled receptor, that is widely expressed, but at particularly abundant levels in kidney, bone, and chondrocytes of the metaphyseal growth plates (6). The human *PTH* gene (chromosome 11p15.3) consists of three exons and two introns. Exon 1 is noncoding, while exon 2 encodes the pre-sequence and part of the pro-sequence. Exon 3 encodes the remaining amino acids of the pro-sequence, the secreted PTH(1-84), and the 3′ untranslated region (7).

Familial isolated hypocalcemia due to insufficient PTH secretion can be caused by several genetic alterations, including activating mutations in the calcium-sensing receptor (CaSR) (8), homozygous or heterozygous mutations in glial cells missing-2 (*GCM2*) (9, 10), heterozygous mutations in *TBX1* (11), or activating Gα11 mutations (*GNA11*) that enhance signaling down-stream of the CaSR (12, 13). Hypocalcemia due to an abnormal PTH, i.e., isolated hypoparathyroidism (IHP), can be caused by homozygous or heterozygous mutations in the *PTH* gene itself. To date, 10 different *PTH* mutations have been reported as causes of IHP (14–16). Six of these mutations are located in exon 2 encoding the pre-sequence, one mutation is located at the splice donor site of exon 2 thus causing abnormal pre-mRNA (pre-messenger ribonucleic acid) splicing, and three mutations are located in exon 3 encoding the secreted PTH(1-84).

Hypocalcemia develops also in the different variants of pseudohypoparathyroidism (PHP), i.e., disorders with PTH-resistance that are caused by inactivating maternal *GNAS* mutations involving the exons encoding the stimulatory G protein (Gsα) (PHP type Ia; PHP1A) or by methylation changes on the maternal *GNAS* allele that reduce Gsα expression (PHP type Ib; PHP1B) (17, 18). Besides mineral ion changes and frequent resistance toward other hormones, patients affected by PHP1A present with developmental features, referred to as Albright Hereditary Osteodystrophy (AHO), which include early-onset obesity, stocky build, neurocognitive abnormalities, brachydactyly, and short stature; such AHO features can also be observed in PHP1B, but they occur less frequently and are typically less severe.

Two recently described sisters (family A) born to consanguineous parents of Middle Eastern descent were found to be hypocalcemic shortly after birth and treatment was implemented to maintain blood calcium levels at the lower end of the normal range (19). After age 30, PTH levels surprisingly increased for both sisters, which, in the absence of AHO, led to the consideration of PHP1B as the underlying disease. One sister, however, had nephrocalcinosis, a frequent finding in hypoparathyroidism (20), but not in PHP1B (21–23). Both patients were eventually found to carry a homozygous PTH variant (A > G change of nucleotide c.94), which changes the first amino acid residue of PTH(1-84) from serine (S1) to proline (P1) (19). The same homozygous S1-to-P1 change was subsequently identified in a third, unrelated patient with early-onset hypocalcemia and elevated PTH levels. We now determined the mechanism through which hypocalcemia develops in patients with this homozygous variant despite an appropriate increase in PTH secretion.

## Results

### Patients with Severe Hypocalcemia and Elevated PTH Levels.

Details of the two patients with hypocalcemia in family A were recently reported (19). The female proband in family B, the first child of first-degree cousins originally from Southern Turkey, presented with seizures because of profound hypocalcemia at the age of 2 mo associated with an elevated PTH level, yet no hypophosphatemia (*SI Appendix*, Fig. S1). She was treated with i.v. calcium and an activated vitamin D analog (alfacalcidol). Other than hypocalcemia, the clinical examination was within normal limits; she had normal cognitive and neuromotor development. During follow-up, her urinary calcium did not exceed the upper end of the age-adjusted normal range because of frequent adjustments of her dietary calcium intake and alfacalcidol doses. Nonetheless, at the age of 6 y, she was noted to have bilateral nephrocalcinosis. Calcium excretion was elevated at 9 y, but within normal limits at 14.6 y. Furthermore, she had several fractures, including an ankle fracture at age 10 and coccyx and knee fractures at age 11. Otherwise, growth and development have been normal (see growth curve in *SI Appendix*, Fig. S1). Upon follow-up serum calcium remained below the normal range, while serum PTH (measured with a commercial intact PTH assay that detects PTH(1-84) and large amino-terminally truncated fragments thereof) increased further and serum phosphate levels remained elevated. Both parents and the two healthy younger sisters revealed normal levels for calcium, phosphate, and PTH.

### Identification of the Previously Reported S1-to-P1 Mutation in PTH(1-84).

**Fig. 1.**
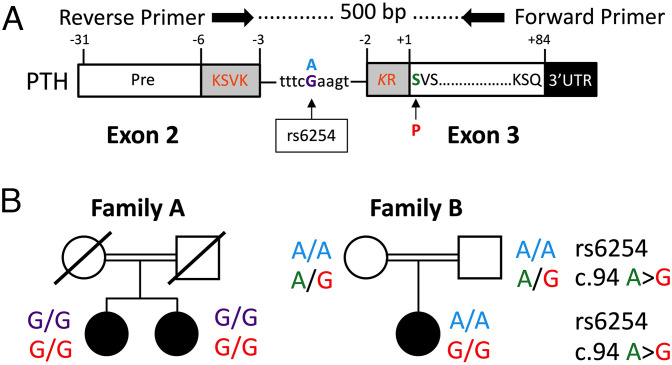
A homozygous Serine-to-Proline mutation affecting the first amino acid of the mature PTH(1-84) in patients with severe hypocalcemia from two unrelated families. (*A*) Schematic presentation of PTH gene and the intervening region. Pre-sequence (white box) and amino acids −6 to −3 of the pro-sequence (gray box) are encoded by exon 2; the remainder of the pro-sequence, the mature PTH(1-84), and the 3′ UTR are derived from exon 3; note that the K at position −2 is shown in italics because one nucleotide encoding this lysine is located in exon 2. Horizontal arrows: forward and reverse primer for amplification of 500 bp across the serine (green S) to proline (red P) mutation and SNP rs6254. (*B*) Pedigrees of families A and B. SNP rs6254 is G/G for the two affected individuals in family A, but A/A for the affected child in family B. The three patients in both families revealed the identical homozygous substitution at nucleotide c.94 A > G that changes serine at position 1 of secreted PTH to proline. Filled black circles, affected females; open circles or squares, unaffected mothers or fathers; line, deceased.

Because of the elevated PTH levels in response to the severe hypocalcemia, a PHP variant was initially considered as the underlying diagnosis. However, nucleotide sequence analysis of the 13 *GNAS* exons encoding Gsα and their splice sites as well as Multiplex Ligation-Dependent Probe Amplification (MLPA) and Methylation-Sensitive MLPA (MS-MLPA) showed no evidence for a genetic or epigenetic *GNAS* defect. Instead, Next Generation Sequencing (NGS) of the patient’s DNA revealed the previously reported homozygous nucleotide transition in *PTH* exon 3 (c.94A > G) (19), which changes S1 of secreted PTH(1-84) to P1. The unaffected parents are both heterozygous c.94A/G (Fig. 1). Notably, the two patients in family A are homozygous G/G for single nucleotide polymorphism (SNP) rs6254, while the index case in family B is homozygous A/A.

### [S1]PTH(1-34) and [P1]PTH(1-34) Induce Cyclic Adenosine Monophosphate (cAMP) Production with Indistinguishable Potency and Efficacy.

**Fig. 2.**
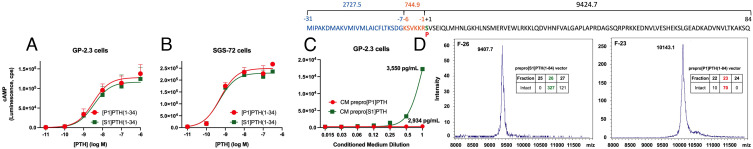
Identification of pro[P1]PTH secreted by COS-7 cells expressing prepro[P1]PTH(1-84). (*A* and *B*) Cyclic AMP formation in GP2.3 cells and SGS-72 cells stimulated with increasing doses of synthetic [S1]PTH(1-34) (green filled squares and lines; EC_50_: 3.5 ± 1.4 nM and 0.5 ± 0.2 nM, respectively) or [P1]PTH(1-34) (red filled circles and lines; EC_50_: 3.2 ± 0.9 nM and 0.5 ± 0.3 nM, respectively). Data are shown as mean ± SEM, n = 3. (*C*) Cyclic AMP formation in GP2.3 cells incubated with increasing concentrations of CM from COS-7 cells expressing prepro[P1]PTH(1-84) (red filled circles and line) or prepro[S1]PTH(1-84) (green filled squares and line). The indicated PTH concentration was measured by intact assay. Data are shown as mean ± SEM, n = 2. (*D*) Affinity and HPLC-purified CM from COS-7 cells transfected with plasmids encoding prepro[S1]PTH(1-84) or prepro[P1]PTH(1-84) was measured by intact PTH assay after the final purification step by HPLC. MassSpec analysis was performed on fractions 23 (*Right*) and 26 (*Left*), respectively. On top: pre-sequence of PTH (2,727.5 Da, blue letters), pro-sequence of PTH (744.9 Da, orange letters), and the sequence of mature PTH (9,424.7 Da, black letters). S1, green letter; P1, red letter.

To determine whether the P1 mutation impairs the biological activity of PTH(1-34), synthetic [S1]PTH(1-34) and [P1]PTH(1-34) were tested in SGS-72 and GP2.3 cells that are derived from human osteosarcoma SaOS-2 and HEK293 cells, respectively (24). Both peptides showed comparable dose-dependent cAMP increases. When using GP2.3 cells, the half-maximal effective concentrations (EC_50_) were 3.5 ± 1.4 nM and 3.2 ± 0.9 nM for [S1]PTH(1-34) and [P1]PTH(1-34), respectively (Fig. 2*A*). Approximately sevenfold higher potencies for cAMP formation were observed with SGS-72 cells (EC_50_: 0.5 ± 0.2 nM and 0.5 ± 0.3 nM for [S1]PTH(1-34) and [P1]PTH(1-34), respectively) (Fig. 2*B*). Maximal second messenger formation was also indistinguishable with both cell lines (GP-2.3 cells: 124,346 ± 29,500 and 137,800 ± 23,317 cpm, respectively; SGS-72 cells: 235,900 ± 3,100 and 267,080 ± 6,000 cpm, respectively, both at 10^−6^ M). These data indicated that the change from serine to proline at position 1 did not alter the biological activity of PTH(1-34). Thus, unless replacement of serine with proline affects the bioactivity of PTH(1-84), but not that of PTH(1-34), it appeared plausible that hypocalcemia despite profoundly elevated PTH levels was caused in the patient in family B, as in the two previously reported cases (19), through a different mechanism.

### Conditioned Medium (CM) from Cells Expressing prepro[P1]PTH(1-84) Fails to Induce cAMP Production.

To determine whether COS-7 cells transfected with plasmids encoding prepro[P1]PTH(1-84) or prepro[S1]PTH(1-84) secrete PTH with similar biological activity, we tested whether CM from these cells increase cAMP accumulation in GP2.3 cells. The PTH concentration in undiluted medium from cells expressing prepro[S1]PTH(1-84) was calculated to be 3,550 pg/mL. This resulted in cAMP formation of 16,600 ± 640 cpm, which is similar to the response to PTH(1-84) at 2.4 × 10^−9^ M. In contrast, undiluted CM from COS-7 cells expressing prepro[P1]PTH(1-84) induced no cAMP response, despite a concentration of immunoreactive PTH of 2,934 pg/mL that was similar to that in CM derived from cells expressing prepro[S1]PTH(1-84) (Fig. 2*C*).

### COS-7 Cells Transfected with the Plasmid Encoding prepro[P1]PTH(1-84) Secrete Only the Mutant Pro-Hormone, Namely pro[P1]PTH(−6 to +84) (pro[P1]PTH).

To determine why COS-7 cells transfected with the plasmid encoding prepro[P1]PTH(1-84) failed to secrete a biologically active peptide, we isolated PTH from CM of COS-7 cells expressing either prepro[S1]PTH(1-84) or prepro[P1]PTH(1-84) for MassSpec analysis using several purification steps that included an immobilized anti-PTH antibody and HPLC; PTH immunoreactivity was assessed after each step by intact PTH assay. When using the medium derived from cells expressing prepro[S1]PTH(1-84), fraction 26 of the final HPLC run contained the highest concentration of PTH (Fig. 2 *D*, *Left*), which revealed a molecular weight of 9,407.7 Da that is consistent with [S1]PTH(1-84) (theoretical MW 9,424.7 Da). In contrast, purified medium from cells expressing prepro[P1]PTH(1-84) showed the highest PTH concentration in HPLC fraction 23, which revealed a molecular weight of 10,143.1 Da (Fig. 2 *D*, *Right*). This PTH variant was 708.4 Da larger than [P1]PTH(1-84) (theoretical MW 9,434.7 Da), suggesting that the pro-sequence of PTH (theoretical MW 744.9 Da) had not been removed and that the mutant pro[P1]PTH was secreted.

### Furin Cleaves [S1]PTH(−6 to +34), but Not [P1]PTH(−6 to +34).

proPTH was previously shown to be cleaved by furin (4, 5). We, therefore, determined whether [S1]PTH(−6 to +34) and [P1]PTH(−6 to +34) can be processed by this enzyme. [S1]PTH(−6 to +34) eluted from the analytical HPLC column at fraction #28 (53% ACN); after incubation with furin, most of the peptide eluted at fraction #29 (55% ACN). MassSpec analysis of furin-treated [S1]PTH(−6 to +34) revealed a single peak with a molecular weight of 4,115.2 Da, which is indistinguishable from that of [S1]PTH(1-34) (theoretical MW: 4,116.8 Da) (*SI Appendix*, Fig. S2 *A* and *C*). In contrast, furin-treated [P1]PTH(−6 to +34) and the untreated peptide eluted from the C18 column at the same fraction (#28; 53% ACN) (*SI Appendix*, Fig. S2 *B* and *D*); furthermore, both peptides revealed indistinguishable molecular weights upon MassSpec analysis (theoretical MW of [P1]PTH(−6 to +34): 4,853.7 Da). These findings suggested that proline at position 1 renders proPTH resistant to cleavage by furin.

### Furin Enhances the Biological Activity of [S1]PTH(−6 to +34), but Not of [P1]PTH(−6 to +34).

**Fig. 3.**
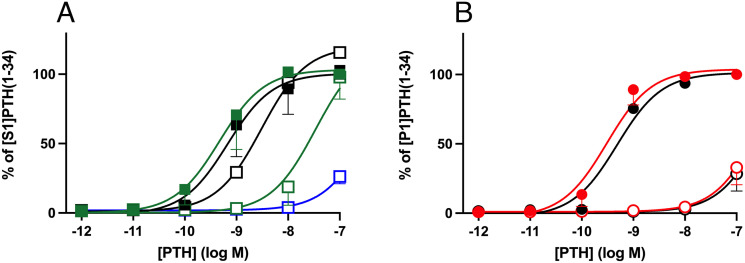
[S1]PTH(−6 to +34), but not [P1]PTH(−6 to +34), is activated by furin. (*A*) Cyclic AMP formation in SGS-72 cells. [S1]PTH(1-34) was incubated in the absence or presence of furin (green filled and black filled squares, respectively; EC_50_: 0.6 ± 0.3 and 0.67 ± 0.9 nM). [S1]PTH(−6 to +34) without addition of furin (green open squares; EC_50_: 16.4 ± 4.7 nM), after incubation with furin (black open squares; EC_50_: 4.3 ± 3.2 nM), or in the presence of a furin inhibitor (Decanoyl-RVKR-CMK) (blue open squares; estimated EC_50_: 271.8 ± 165.7 nM). Data are shown as mean ± SEM, n = 2; error bars are often smaller or similar than the sizes of the symbols. (*B*) Cyclic AMP formation in SGS-72 cells. [P1]PTH(1-34) was incubated in the absence or presence of furin (red filled or black filled circles, respectively; EC_50_: 0.3 ± 0.09 and 0.5 ± 0.2 nM). [P1]PTH(−6 to +34) with or without furin (black open or red open circles, respectively; estimated EC_50_: 370.8 ± 73.8 and 305.3 ± 33.8 nM). EC_50_ of [P1]PTH(−6 to +34) with or without furin, [S1]PTH(−6 to +34) with furin inhibitor and [S1]PTH(−6 to +34) without furin were estimated by constraining the top of each curve to the maximum cAMP formation by PTH(1-34) at 10^−7^ M. Data are shown as mean ± SEM, n = 4.

The biological activity of [S1]PTH(−6 to +34) in SGS-72 cells was more than an order of magnitude lower than that of [S1]PTH(1-34) (EC_50_: 16.4 ± 4.7 vs. 0.6 ± 0.3 nM), and it was even lower when the reporter assay was performed in the presence of decanoyl-RVKR-chlororomethylketone (CMK), a furin inhibitor, to reduce the intrinsic activity of furin enzyme produced by the cells (estimated EC_50_: 271.8 ± 165.7 nM) (Fig. 3*A*). This suggested that our reporter cells express this proprotein convertase on the cell surface or release the enzyme into the extracellular space, as described (25). Furin treatment did not alter the biological activity of [S1]PTH(1-34). However, by removing the pro-sequence and thus generating [S1]PTH(1-34) (*SI Appendix*, Fig. S2 *A* and *C*), the enzyme decreased the EC_50_ for cAMP formation by [S1]PTH(−6 to +34) from 16.4 ± 4.7 to 4.3 ± 3.3 nM. Furin-treated [S1]PTH(−6 to +34) thus induced a dose-dependent cAMP increase, which was similar to that of [S1]PTH(1-34) [maximum at 10^−7^ M: [S1]PTH(−6 to +34) was 115.7 ± 8.6% compared to [S1]PTH(1-34)].

Similar to [S1]PTH(−6 to +34), the potency of cAMP formation induced by [P1]PTH(−6 to +34) was lower than that of [P1]PTH(1-34) (estimated EC_50_: 305.3 ± 33.8 nM vs. 0.30 ± 0.1 nM). Furin treatment had no effect on the biological activity of [P1]PTH(1-34) or [P1]PTH(−6 to +34) (Fig. 3*B*).

### An Immunoassay Detects pro[P1]PTH but Shows Almost No Cross-Reactivity with PTH(1-84).

Serial dilutions of CM from COS-7 cells transfected with the plasmid encoding prepro[P1]PTH(1-84) run in parallel to the PTH(1-84) standard curve, when measured by the intact PTH assay. The concentration of pro[P1]PTH in undiluted CM was calculated to be 4,825 pg/mL and aliquots thereof were stored at −20 °C for use as standard in all subsequent immunoassays.

**Fig. 4.**
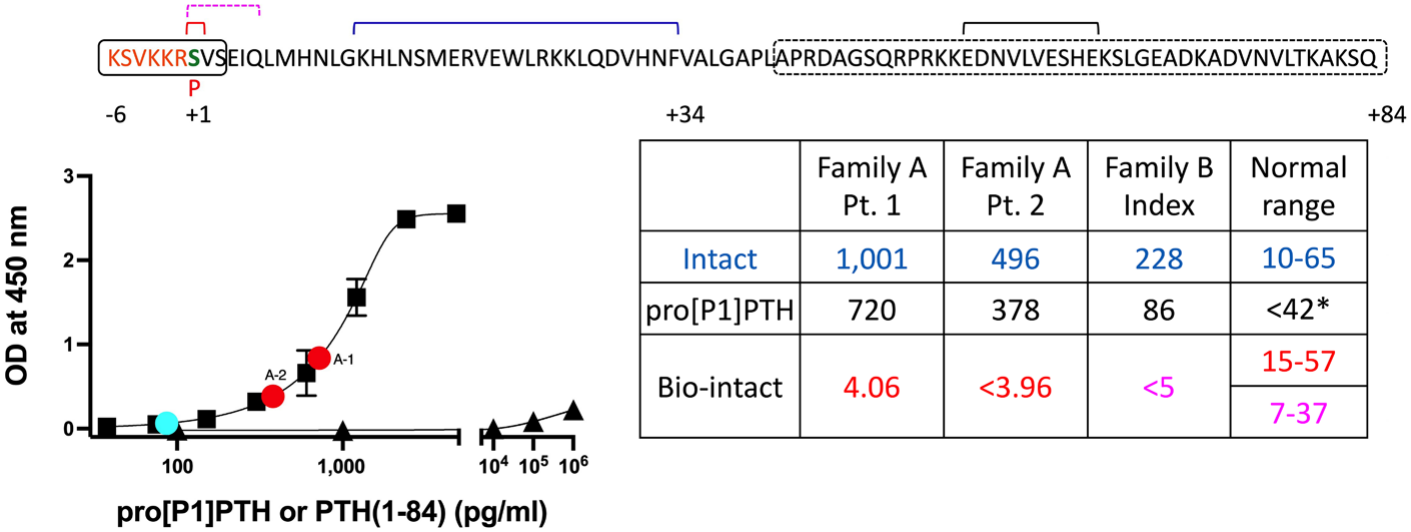
Detection of pro[P1]PTH in plasma of patients with homozygous S1-to-P1 mutations. COS-7 cells were transfected in a T175 flask with the plasmid encoding prepro[P1]PTH(1-84). The PTH concentration in the CM from these cells was 4,825 pg/mL, as determined by the intact PTH assay. To establish an assay for the measurement of pro[P1]PTH, the detection antibody in the intact PTH assay was replaced with the anti-pro[P1]PTH^HRP^ antibody that had been purified with immobilized pro[P1]PTH(−6 to +3) (black rectangle). Using serial dilutions of CM (black filled squares), the concentration of pro[P1]PTH was estimated for three patients who are homozygous for the P1 mutation (red and blue filled circles); the lower limit for detection of pro[P1]PTH was 42 pg/mL; 10 healthy controls had undetectable levels in this PTH assay. Detection of PTH(1-84) (black filled triangles) was minimal even at very high concentrations. Table: PTH levels as measured by three different PTH assays; normal ranges are indicated. The proPTH sequence is shown above; brackets and rectangles indicate the epitopes recognized by the different anti-PTH antibodies. Intact PTH assay (Immutopics; measurement results in blue): capture antibody directed against amino acid residues 39 to 84 (black stippled rectangle); detection antibody directed against amino acid residues 13 to 34 (blue bracket). Bio-intact PTH assay (Roche; measurement results in red): capture antibody directed against amino acid residues 1 (red bracket); detection antibody directed against amino acid residues 55 to 64 (black bracket). Bio-intact PTH assay (DiaSorin; measurement results in purple): capture antibody against amino acid residues 39 to 84 (black stippled rectangle), detection antibody amino acid residues 1 to 6 (purple bracket). *; limit of detection of the pro[P1]PTH Enzyme-Linked ImmunoSorbent Assay (ELISA).

To allow quantification of pro[P1]PTH in CM and in plasma of our patients with the homozygous P1 mutation, we replaced the detection antibody in an intact PTH assay (Immutopics) with a horseradish peroxidase (HRP)-labeled antibody raised against pro[P1]PTH(−6 to +15), which was affinity purified with pro[P1]PTH(−6 to +3) (anti-pro[P1]PTH^HRP^ antibody) (*Materials and Methods*). Binding of the anti-pro[P1]PTH^HRP^ detection antibody increased progressively as increasing concentrations of pro[P1]PTH were bound to the capture antibody directed against PTH(39-84); the lower limit of detection for pro[P1]PTH was 42 pg/mL and half maximum binding of the anti-pro[P1]PTH^HRP^ detection antibody was at 1,032 pg/mL (Fig. 4). PTH(1-84) was barely detected indicating that our pro[P1]PTH assay can readily distinguish between pro[P1]PTH and PTH(1-84). While pro[P1]PTH was undetectable in plasma from controls, plasma from the three patients with the homozygous P1 mutation had levels of 720, 378, and 86 pg/mL, respectively, which are 28%, 24%, and 62%, respectively, lower than the concentrations measured in the intact PTH assay (Fig. 4, Table). When measured with biointact PTH assays, the levels were undetectable or well below the lower end of the normal range for the three patients with a homozygous P1 mutation.

### Characterization of the Epitope Detected by the Anti-[P1]PTH(−6 to +3) Antibody.

For additional characterization of our anti-[P1]PTH(−6 to +3) antibody, we biotin-labeled this antibody for use as capture antibody (anti-[P1]PTH(−6 to +3)^Biotin^ antibody) in combination with the HRP-labeled detection antibody from the intact PTH assay (Immutopics) that is directed against PTH(13-34) (*SI Appendix*, Fig. S3). When using these reagents, [S1]PTH(1-34) was barely detectable, while the detection of pro[P1]PTH was equivalent to that observed when using the anti-[P1]PTH(−6 to +3)^HRP^ antibody for detection (half-maximal binding: 1,330 pg/mL; lower limit of detection: 37 pg/mL).

Given that capture and detection antibodies in this assay are directed against different portions of PTH(−6 to +34), we were able to partially define the epitope recognized by the anti-[P1]PTH(−6 to +3)^Biotin^ antibody. [P1]PTH(−6 to +34) and [S1]PTH(−6 to +34) were detected equivalently, while stepwise removal of the amino-terminal amino acid residues from [S1]PTH(−6 to +34) resulted in progressively reduced cross-reactivity. [S1]PTH(1-34) and PTH(2-34) showed only minimal cross-reactivity at very high concentrations, while PTH(3-34) and PTH(4-34) were not detected at all. After incubation of [S1]PTH(−6 to +34) with furin to remove residues −6 to −1, detection was reduced by at least 10-fold, while detection of furin-treated [P1]PTH(−6 to +34) remained unchanged. Additional characterization indicated that the epitope recognized by the anti-[P1]PTH(−6 to +3)^Biotin^ antibody resides predominantly in the −6 to −4 region.

## Discussion

We reported a patient, who had initially presented with severe, neonatal hypocalcemia due to the same homozygous S1-to-P1 mutation of the mature secreted PTH that had previously been identified in two sisters (19) but could not be found among publically available databases (ClinVar, gnomAD, and 1,000 genomes). Although originally from the same geographic area in the Middle East, the disease-causing nucleotide change (A > G) had most likely emerged independently in both families because the patients in family A revealed different homozygous nucleotides at SNP rs6254 than the patients in family B, namely G/G vs. A/A. This raises the possibility that a heterozygous S1-to-P1 change can be found at a presumably low frequency in the general population, at least in Middle Eastern countries.

Surprisingly, [S1]PTH(1-34) and [P1]PTH(1-34) revealed indistinguishable biological activity when tested with SGS-72 and GP2.3 cells. CM from cells expressing prepro[P1]PTH(1-84), however, had no biological activity when tested on GP2.3 cells, while CM from cells expressing prepro[S1]PTH(1-84) led to the expected dose-dependent increase in cAMP formation. This raised the possibility that the S1-to-P1 change impairs intracellular processing of preproPTH. In fact, stepwise purification of CM from COS-7 cells expressing the prepro[P1]PTH(1-84) variant, followed by MassSpec analysis, revealed secretion of pro[P1]PTH. In contrast, CM from cells expressing wild-type prepro[S1]PTH(1-84) allowed purification of [S1]PTH(1-84), but no pro[S1]PTH(1-84) was identified. Even though cleavage prediction algorithms suggested that furin-dependent cleavage efficiency of the hormonal precursor is only partially impacted by the P1 mutation (*SI Appendix*, Fig. S4), our experimental data indicated that the amino acid change strongly impeded furin-dependent cleavage of the six amino acid pro-sequence, presumably due to a change in primary and/or secondary structures.

Cleavage of the six amino acid pro-sequence of PTH was previously shown to be mediated by furin (4, 5). Consistent with these earlier findings, [S1]PTH(−6 to +34) was cleaved in our experiments by recombinant furin to generate biologically active [S1]PTH(1-34); in contrast, [P1]PTH(−6 to +34) failed to be processed by this proprotein convertase. Interestingly, incubation of [S1]PTH(−6 to +34) with a furin-specific inhibitor reduced generation of the biologically active PTH(1-34) indicating that furin is present on the surface of our SGS-72 reporter cells; alternatively, the enzyme is secreted into the medium, as described (25). Consistent with this conclusion, COS-7 cells were able to generate [S1]PTH(1-84) when transfected with the plasmid encoding prepro[S1]PTH(1-84) (Fig. 2). Likewise, CM from GH4C1 cells infected with a vector encoding human preproPTH had previously revealed that PTH(1-84), but not proPTH, can be detected by Western blot analysis using an antibody raised against [S1]PTH(−6 to +7) (5). This indicates that the generation of PTH(1-84) from an unmodified PTH precursor by furin or other proprotein convertases is highly efficient in different cell types.

Several experimental approaches showed that proline instead of serine at position 1 fails to generate biologically active PTH by preventing the removal of the six amino acid pro-sequence. It remains to be determined, however, whether amino acid residues other than proline at position 1 can also impair the processing of proPTH. Gardella et al. had previously shown that substitution of S1 with proline, as well as valine, tyrosine, or asparagine resulted in little or no biologically active PTH in a CM of transfected COS-7 cells, but it is unclear whether the effects of the other residues could be explained by the amino acid changes themselves, by impaired secretion, or by altered proPTH cleavage (26). Interestingly, [P1]PTH(−6 to +34) showed approximately 100-fold lower cAMP formation than [S1]PTH(1-34) or [P1]PTH(1-34). It is therefore conceivable that the proPTH mutant, if it were to be secreted at high enough concentrations, could contribute some to calcium and phosphate homeostasis in the patients.

To determine whether pro[P1]PTH is present in the circulation of patients with the homozygous P1 variant, we generated an HRP-labeled antibody, which was affinity purified with immobilized pro[P1]PTH(−6 to +3), and used it in place of the detection antibody in a commercially available assay kit (27). The resulting modified PTH assay allowed the sensitive detection of pro[P1]PTH secreted by COS-7 cells, which had been transfected with the prepro[P1]PTH(1-84) plasmid; compared to pro[P1]PTH, cross-reactivity with PTH(1-84) was extremely low. Using this in-house assay, we showed that plasma samples from healthy controls had undetectable levels of pro[P1]PTH, while the three patients with the homozygous P1 variant had readily detectable levels of the pro-hormone. Importantly, for the two patients in family A, the concentrations of pro[P1]PTH were approximately 28% and 24% lower than those detected with an intact assay indicating that most of the circulating PTH are pro[P1]PTH in both patients. Although synthetic pro[P1]PTH(−6 to +84) is not available for rigorous comparisons, dilutions of pro[P1]PTH and PTH(1-84) run in parallel, making it likely that pro[P1]PTH and the mature secreted hormone are detected equivalently by the intact PTH assay. It is therefore conceivable that any differences between the concentrations of PTH detected in the pro[P1]PTH and the intact PTH assay could be due to the presence of non-(1-84)PTH fragments, such as PTH(7-84), that are as readily detected as PTH(1-84) by the intact PTH assay (28), but not by our new pro[P1]PTH assay. For the pediatric patient in family B, only about one-third of the PTH measured by the intact assay appears to be pro[P1]PTH. This could indicate that cleavage of the mutant pro-hormone to putative non-(1-84)PTH fragments is less efficient than in the two older patients, who developed severe parathyroid hyperplasia and thus much higher circulating PTH levels. Alternatively, enzymes other than furin may be able to process pro[P1]PTH(−6 to −84) into [P1]PTH(1-84), possibly only at a younger age.

The findings related to the S1-to-P1 change seem opposite to those observed for three siblings (29) and a recently described unrelated patient (30), who presented with severe hypocalcemia due to a homozygous [R25C]PTH mutation. This latter amino acid change renders PTH(1-84) much less active in vivo, thus explaining the mineral ion abnormality encountered in these patients. The levels of immunoreactive PTH in these patients were low when measured with the intact PTH assay that uses a detection antibody directed against the PTH(13-34) region, yet profoundly elevated when measured with a bio-intact assay using a detection or capture antibody directed against PTH(1-3) (29, 30). As for our patients with the homozygous P1 mutation, hypocalcemia due to the mutant PTH could have led to the diagnosis of either hypoparathyroidism or PHP, depending on the assay used for PTH measurement. Given that hypocalcemia is due to a secreted pro-hormone with much reduced biological activity rather than PTH-resistance, it is conceivable that patients with homozygous mutation of either amino acid residue S1 or R25 would benefit from continuous treatment with PTH(1-34) or a long-acting PTH analog (31–33). Such interventions might allow maintaining higher blood calcium levels without causing hypercalciuria, thereby preventing parathyroid hyperplasia and possibly parathyroid adenomas or even malignancies.

The two affected sisters in family A were treated with doses of oral calcium and vitamin D analogs that were appropriate to maintain serum calcium levels at the lower end of the normal range, thereby preventing symptomatic hypocalcemia. However, because of low-normal blood calcium levels, both patients, who have by now been followed for more than 50 y, gradually developed secondary hyperparathyroidism resulting in considerable enlargement of all four parathyroid glands. However, parathyroid hyperplasia was not caused by enhanced intracellular accumulation of the mutant pro[P1]PTH, as in dominant variants of hypoparathyroidism (34, 35) but because of hypocalcemia and consequently insufficient activation of the CaSR. The mutant pro-hormone was readily secreted in vitro and in vivo. Consequently, plasma PTH concentrations rose to levels that were up to 100 times above the upper end of the normal range. We have now determined that these much elevated PTH levels comprise to a considerable extent the pro-hormone, which activates the PTHR1 with much reduced efficiency. This raises the possibility that the C-terminal portion of PTH is unlikely to play a major role in the direct regulation of mineral ion homeostasis. The previously characterized receptor/binding protein that interacts with PTH(19-84) or a similar PTH fragment (36, 37) may thus mediate as-yet undefined biological activities, some of which could affect calcium regulation indirectly (28, 38, 39).

It remains to be determined whether PTH mutations other than P1 can affect intraglandular processing of proPTH. Furthermore, rare heterozygous variants replacing the first amino acid or an adjacent residue in the pro-sequence or the mature peptide have been identified in the precursors of several different peptide hormones (*SI Appendix*, Table S1). It is therefore plausible that impaired pre- or pro-sequence cleavage leads to abnormal biological activity of other hormones, cytokines, growth factors, or other secreted or membrane-embedded proteins if the nucleotide changes altering these amino acids are present on one or both alleles. Our findings may thus have broader implications for numerous other regulatory systems.

## Materials and Methods

### NGS, MLPA, MS-MLPA, and PCR Amplification of Genomic DNA.

Targeted NGS was performed with MiSeq (Illumina) and MLPA/MS-MLPA was performed as described ((40) and *SI Appendix*). To genotype SNP rs6254 and to confirm the [P1]PTH(1-84) mutation, genomic DNA was PCR-amplified using forward primer 5′-TTCATGGCTCTCAACCAAGACA-3′ and reverse primer 5′-AAGCTTCTCGTGAAAACCAACC-3′. Cycling conditions: 95 °C, 5 min; 35 cycles of 95 °C, 20 s; 58 °C, 15 s; 72 °C, 25 s; final extension at 72 °C, 10 min.

### Peptide Synthesis.

All peptides were synthesized with a C-terminal amide (NH_2_) instead of free carboxylic acid (COOH) by the Massachusetts General Hospital (MGH) Peptide/Protein Core Facility, these included: [P1]PTH(−6 to +34), [S1]PTH(−6 to +34), [S1]PTH(−5 to +34), [S1]PTH(−4 to +34), [S1]PTH(−3 to +34), [S1]PTH(−2 to +34), [S1]PTH(−1 to +34), [S1]PTH(1-34), [P1]PTH(1-34), pro[P1]PTH(−6 to +3) (coupled to a hydrophobic spacer at the C-terminus followed by a cysteine), PTH(2-34), PTH(3-34), and PTH(4-34) as well as pro[P1,W14,C15]PTH(−6 to +15) [pro[P1]PTH(−6 to +15)] for coupling to Keyhole Limpet Hemocyanin (KLH) (pro[P1]PTH(−6 to +15)-KLH) (*SI Appendix*). Recombinant PTH(1-84) was from Chugai Pharmaceutical Co., Ltd. Peptide quality was verified by analytical HPLC and mass spectrometry (MassSpec).

### PTH Expression Vectors and Mutagenesis.

P1 was introduced into the plasmid encoding wild-type human prepro[S1]PTH(1-84), as described (29) to encode prepro[P1]PTH(1-84).

### Cell Culture.

COS-7 cells were cultured in Dulbecco's Modified Eagle’s Medium (DMEM) (10% fetal bovine serum (FBS), 1%  penicillin/streptomycin). After reaching confluency, cells were lifted with 0.1% trypsin-EDTA and the single-cell suspension was transfected in a 6-well plate or in a T175 flask with plasmids encoding either prepro[S1]PTH(1-84) or the prepro[P1]PTH(1-84) mutant using Lipofectine 2000 (ThermoFisher Scientific), as described (41). Forty-eight hours after transfection, the medium was replaced with DMEM without FBS. The CM was collected daily for another 3 d and stored at −80 °C until further experimentation.

### PTH-Stimulated cAMP Formation Using SaOS-2 and HEK293 Reporter Cells Expressing the Human PTHR1.

SGS-72 cells, i.e., human osteosarcoma SaOS-2 cells stably expressing the Glosensor cAMP reporter, were maintained in modified McCoy’s 5A medium [15% FBS, 1% penicillin/streptomycin, and 1% nonessential amino acids, as described (29)]. GP2.3 cells, i.e., HEK293 cells stably expressing the human PTHR1 and the Glosensor cAMP reporter, were maintained in DMEM (10% FBS, 1% penicillin/streptomycin), as described (24). Cells were seeded into 96‐well white plates (40,000 cells/100µL/well); 24 to 48 h after confluency, the wells were rinsed with 100 µL carbon dioxide-independent culture medium (CIDB; Life Technologies) containing 0.1% bovine serum albumin (BSA), which was then replaced with 90 µL of luciferin (0.5 mM in CIDB) for 15 min before adding 10 µL of increasing concentrations of PTH analogs diluted in CIDB or 10 µL of CIDB, and then measuring cAMP‐dependent luminescence at 2‐min intervals using a PerkinElmer Envision plate reader (PerkinElmer), as described (24). For each well, the maximum luminescence (counts per second, cps) was typically observed 20 to 23 min after ligand addition, which was used to generate ligand dose–response curves, as described (24). GP2.3 cells were also used for the evaluation of CM from transfected COS-7 cells; PTH(1-84) served as control.

### Generation of Affinity-Purified Antibody Against pro[P1]PTH.

Two rabbits were immunized at Cocalico Biologicals, Inc. with pro[P1]PTH(−6 to +15)-KLH emulsified with Freund’s complete adjuvant. After several booster injections emulsified with Freund’s incomplete adjuvant, antibody titers were evaluated using standard procedures, as described (42). In brief: 100 μL of pro[P1]PTH(−6 to +3) (10 μg/mL in PBS) was added to 96 wells (Greiner Bio-One MICROLON™, Fisher Scientific) for 3 h at 37 °C. Diluted rabbit antisera [100 μL/well; 1:100 to 1:1,000,000 in PBS/0.1% BSA (Bovine Serum Albumin)] or PBS/0.1% BSA (30 min, 37 °C) was added after several washes. Horseradish peroxidase (HRP)-labeled goat anti-rabbit IgG (Abcam, Cambridge, UK; 0.02 µg/mL in PBS/0.1% BSA; 100 μL/well) was used (30 min, 37 °C) for detection. Absorbance was read at 450 nm on a Envision plate reader (Perkin Elmer).

### Antibody Purification, HRP-, and Biotin-Conjugation.

pro[P1]PTH(−6 to +3) was conjugated to SulfoLink coupling gel via the C-terminal cysteine (PIERCE) for affinity purification of the rabbit antibodies, which were then HRP-labeled using EZ-Link Plus Activated Peroxidase Kit (Thermo Fisher Scientific), following the manufacturer’s protocol. The anti-pro[P1]PTH(−6 to +3) antibody was also conjugated to biotin using EZ-LinkTM Sulfo-NHS-LC-Biotinylation Kit (Thermo Fisher Scientific), following the manufacturer’s protocol, except using 40-fold instead of 20-fold excess of biotin. After biotin coupling, 15% of rabbit serum were added to the biotinylated antibody [anti-pro[P1]PTH(−6 to +3)^Biotin^].

### PTH Measurements in Patients’ Plasma.

Three different Enzyme-Linked ImmunoSorbent Assay (ELISA) kits for PTH measurements were used; an intact PTH assay (Immutopics Inc.) that detects PTH(1-84) and large amino-terminally truncated PTH fragments and two-third generation PTH assays, which use antibodies directed against the first two or six amino acid residues for either detection or capture thus measuring only biointact PTH(1-84). The assay from Roche was used for the index case in Family B, and the assay from DiaSorin (The Liaison (1-84) PTH immunoassay) was used for both patients in Family A (27, 43–45). For development of an assay that measures pro[P1]PTH(−6 to +84) (pro[P1]PTH) without significant PTH(1-84) cross-reactivity, reagents of the intact assay (Immutopics) were used, except that the detection antibody was replaced with our affinity-purified, HRP-labeled antibody raised against pro[P1]PTH(−6 to +3) (anti-pro[P1]PTH^HRP^ antibody).

### Purification of PTH from CM.

Sep-Pak C18 cartridges were rinsed with 100% ACN/0.01% Trifluoroacetic Acid (TFA) (2 × 5 mL), followed by equilibration with H_2_O/0.01% TFA (5 × 5 mL) before loading with CM without FBS (180 mL) from COS-7 cells transfected with plasmids encoding prepro[S1]PTH(1-84) or prepro[P1]PTH(1-84). Elution was performed sequentially with 5 mL of 50%, 75%, and 100% ACN/0.01% TFA. The eluates were lyophilized and reconstituted in 2 mL of 30% ACN/0.01% TFA. HPLC was then performed on the partially purified CM using a C18 column (4.6 mm × 250 mm; Vydac, Avantor). Then, 2 mL of partially purified wild-type or mutant PTH were loaded into a 2-mL loop. After injection, the column was equilibrated for 5 min with 5% ACN/0.05% TFA, before applying a gradient of 25 to 55% of ACN/0.05% TFA over 30 min at a flow rate of 1 mL/min. The two fractions with the highest PTH concentration [derived from cells expressing either prepro[S1]PTH(1-84) or prepro[P1]PTH(1-84)] were pooled and incubated with 500 µL of biotinylated anti-PTH(39-84) antibody (Immutopics) (2 h, room temperature) before adding streptavidin beads (300 µL; 1 h, room temperature). Beads were then rinsed twice with phosphate-buffered saline (PBS) and the captured PTH was eluted with 300 µL of glycine-HCl, pH 2.8. Eluted samples were mixed with 700 µL of 30% ACN/0.01% TFA.

A second HPLC was performed on a C4 column (2.1 mm × 150 mm; DAISO Industries Co., Ltd.). Then, 1 mL of either affinity purified wild-type or mutant PTH was loaded into a 2 mL loop. After injection, the column was equilibrated for 5 min with 5% ACN/0.05% TFA, before applying a gradient of 30 to 60% of ACN/0.05% TFA over 30 min at a flow rate of 0.5 mL/min. Then, 5 µL of each fraction was measured by intact PTH ELISA. The fractions with the highest concentration were concentrated by speedvac to a volume of 2 µL. MassSpec analyses using 1 µL of the concentrated fractions was performed on a microflex instrument (Bruker), as described (46).

### Assessment of PTH Analogs Cleavage by Furin.

#### Furin incubation followed by HPLC analysis and MassSpec.

[S1]PTH(−6 to +34) or [P1]PTH(−6 to +34) (10 µg/10 µL 10 mM acetic acid) was incubated with furin (8U/4 μL) (New England Biolabs) and furin buffer (50 mM HEPES (4-(2-hydroxyethyl)-1-piperazineethanesulfonic acid)–KOH (potassium hydroxide) (pH 7.4), 150 mM NaCl, 5 mM MgCl_2_, 16 mM imidazole, 2% glycerol, 0.15 mM EGTA, 500 μM ATP, 0.5% Triton-X100, 2 mM CaCl_2_, and 2 mM β-mercaptoethanol) (RT, 60 min; final volume 30 µL) (New England Biolabs), as recommended by the manufacturer, before adding EGTA (100 μM, 3 μL). In some experiments, 1 μL of the reaction buffer was replaced with the furin inhibitor Decanoyl-RVKR-CMK (Tocris Bioscience; 50 μM in water). To assess cleavage, 5 µL of furin-treated or untreated peptides (1.5 µg each) was loaded onto a C18 column (2.1 mm × 150 mm, Higgins Analytical, Inc.), and a gradient of 4 to 76% ACN/0.05% TFA over 40 min (200 µL/min) was applied. To determine the molecular weight, 10 µL of furin-treated or untreated peptides was loaded onto a C18 ZipTip (Millipore Corporation) that had been rinsed with 100% ACN/0.01%  TFA (2 × 20 μL) followed by equilibration with H_2_O/0.01% TFA (5 × 20 μL). Peptide elution with 100% ACN/0.01% TFA was followed by speed vac and MassSpec analysis.

#### In vitro  activity.

SGS-72 cells were used for assessing PTH-stimulated cAMP formation. 10 µL of increasing concentrations of [S1]PTH(−6 to +34) or [P1]PTH(−6 to +34) before and after incubation with furin were diluted in CIDB buffer. In addition, 3 μL of Decanoyl-RVKR-CMK (50 μM dissolved in water) were added into wells with [S1]PTH(−6 to +34) to assess cleavage inhibition that occurred in SGS-72 cells. The results are expressed as percentage of [S1]PTH(1-34) or [P1]PTH(1-34).

### Data Processing and Statistical Analyses.

Prism 9.0 (GraphPad Software Inc.) was used for data processing as described in ref. (24).

## Supplementary Material

Appendix 01 (PDF)

## Data Availability

All study data are included in the article and/or *SI Appendix*.
