# Supplementary material for: Homozygous Ser-1 to Pro-1 mutation in parathyroid hormone identified in hypocalcemic patients results in secretion of a biologically inactive pro-hormone
Source: Proc Natl Acad Sci U S A. 2023 Feb 16;120(8):e2208047120. doi: 10.1073/pnas.2208047120 (PMC9974466; doi:10.1073/pnas.2208047120)
Supplement: Supplementary file 1 — Appendix 01 (PDF) [file pnas.2208047120.sapp.pdf]

Supplementary Information for

Homozygous Ser-1 to Pro-1 mutation in parathyroid hormone identified in hypocalcemic patients results in secretion of a biologically inactive pro-hormone.

Patrick Hanna, Ashok Khatri, Shawn Choi, Severine Brabant, Matti L. Gild, Marie L. Piketty, Bruno Francou, Dominique Prié, John T. Potts Jr, Roderick J. Clifton-Bligh, Agnès Linglart, Thomas J. Gardella, and Harald Jüppner

Corresponding author: John T. Potts, Jr., MD
Endocrine Unit, Thier 10, 50 Blossom St., Massachusetts General Hospital, Boston, MA 02114, USA
jtpotts@mgh.harvard.edu

This PDF file includes:

Supplementary text for Materials and Methods
Figures S1 to S4
Table S1

SUPPLEMENTARY INFORMATION TEXT

Next Generation Sequencing (NGS)

DNA was isolated from 5 mL of whole peripheral blood (QIAGEN DSP DNA Midi Kit). Whole genome Illumina libraries were prepared from genomic DNA using NEBNext® UltraII FS DNA Library Prep Kit for Illumina® (NEB Inc.) based on enzymatic fragmentation and automated on a Biomek Span 8 workstation (Beckman). Illumina adapters and other oligonucleotides were provided by Eurofins Genomics. Enrichment was performed using a Sure Select XT kit (Agilent) automated on a Biomek 4000 workstation (Beckman). The panel of genes was designed using the Agilent Sure Design web application (Agilent) and is available upon request. Paired-end Sequencing (2*150 bp) was performed on NextSeq550 or MiSeq. The identification of single nucleotide variants (SNV) and small indels (up to 50bp) mutations was carried out automatically by the Galaxy Bioinformatics Platform of APHP Paris-Saclay University Hospital. Readings of the Fastq end pairs were taken after alignment of the sequences to the reference human genome 19 (hg19) using the BWA-MEM 0.7.10 software. Variant selection was done using GATK 3.4-46. The results were confirmed by Sanger sequencing on both strands in independent experiments.

Peptide synthesis

Peptide analogs were synthesized on an automated Peptide Synthesizer (model Apex 396; AAPPTec, Louisville, KY) with Fmoc/tBu solid-phase chemistry using *N,N*-Diisopropyl Carbodiimide (DIC)/1 – Hydroxybenzotriazole (HOBt) activation (1). Dried peptide resins were cleaved with a cleavage mixture K, containing TFA/thioanisole/water/-phenol/ethanedithiol (82.5:5:5:5:2.5 v/v) for 3 h at room temperature (2). Crude peptides were precipitated and washed twice in cold methyl tert-butyl ether (MTBE). Precipitates were then dissolved in 20% ACN/0.1% TFA in dH_2O and freeze-dried.

Peptide purification

Crude peptides were dissolved in 20% ACN/0.1% TFA in dH_2O and purified on a 250 × 20 mm reversed-phase column (Higgins Analytical Targa C18 Semi-Preparative) and a Waters HPLC using a 20–40% B gradient in 20 min at a flow rate of 15 ml/min. Buffer A was 0.1% TFA in dH_2O and Buffer B was 0.1% TFA in ACN. Approximately 3.0 ml fractions were collected manually and then analyzed on a Microbore HPLC (Applied Biosystems, model 120A) using a buffer system consisting of Buffer A: 0.06% TFA/dH_2O and Buffer B: 0.05% TFA in 80% ACN/20% dH_2O; a reversed-phase 2.1 × 150 mm column (Vydac C18) with a flow rate of 400 µl/min using a gradient of 5–95% B over 9 min. Fractions containing the purified peptides were pooled and freeze-dried.

Peptide characterization by Mass Spectrometry

Peptides/Proteins were analyzed by matrix-assisted laser desorption ionization/desorption time of flight mass spectrometry (MALDI-TOF-MS) (3) on a Bruker, Microflex instrument using alpha-cyano-4-hydroxycinnamic acid matrix.

SUPPLEMENTAL FIGURES AND TABLE

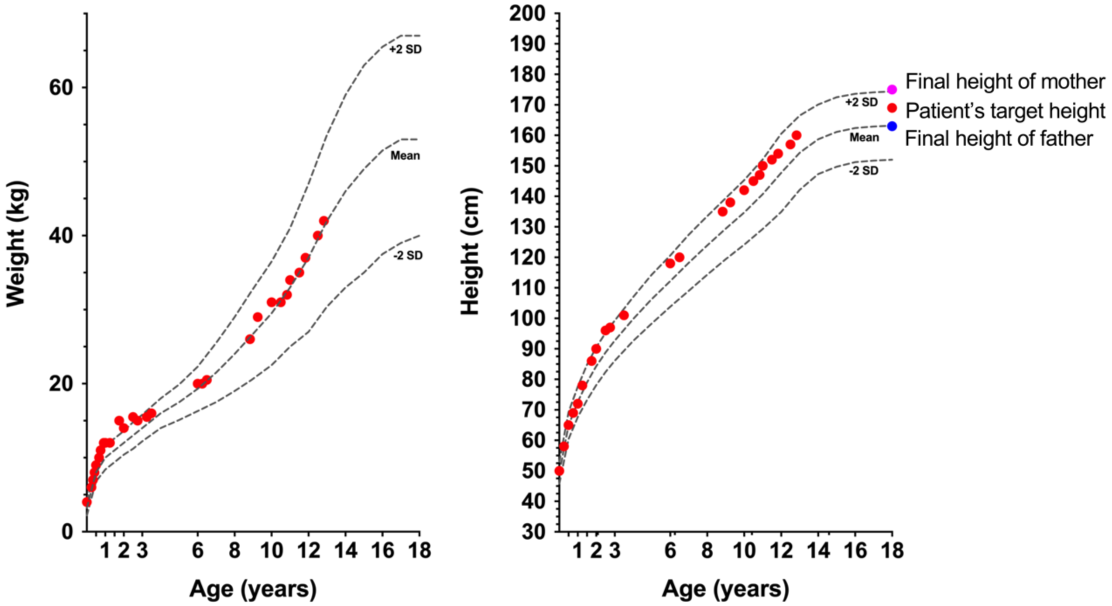

	2 months of life	9 years	14.6 years
Calcium (2.20-2.60 mmol/l)	1.17	2.12	2.10
Phosphate	1.65 (1.55-2.39 mmol/l)	1.83 (1.16-1.87 mmol/l)	2.16 (0.74-1.45 mmol/l)
PTH (10-50 pg/ml)	110	472	359
25OHD (30-60 ng/ml)			21
Urinary calcium		5 mg/kg/day (0.44-3.28)	0.1 calcium/creatinine (0.04-0.7 mmol/mmol)

Fig. S1:

Laboratory data and growth.

Serum calcium, phosphate, PTH, 25OH vitamin D levels, and urinary calcium excretion at different ages (Table), as well as growth and weight curves for the patient in family B.

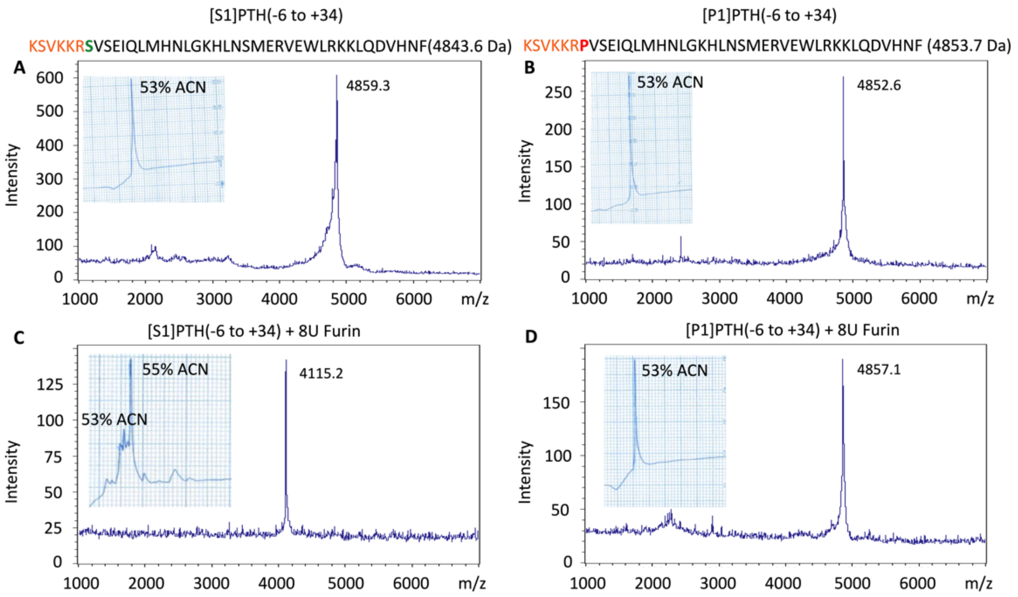

Fig. S2:

[S1]PTH(-6 to +34), but not [P1]PTH(-6 to +34), is cleaved by furin. All peptides were synthesized with a C-terminal NH_2 instead of COOH.

Panels A and C: HPLC and Mass spectrometric analyses of [S1]PTH(-6 to +34) before and after incubation with furin. HLPC: 4-76% ACN/0.05% TFA over 40 minutes (200 µl/min). [S1]PTH(-6 to +34) eluted at 53% ACN and [S1]PTH(1-34) eluted at 55% ACN. Mass spectrometric analyses revealed single peaks at 4859.3 Da for [S1]PTH(-6 to +34) and at 4115.2 Da for [S1]PTH(-6 to +34) after incubation with furin.

Panels B and D: HPLC and Mass spectrometric analyses of [P1]PTH(-6 to +34) before and after incubation with furin. HLPC: 4-76% ACN/0.05% TFA over 40 minutes (200 µl/min). [P1]PTH(-6 to +34) eluted at 53% ACN; incubation with furin did not change the molecular weight.

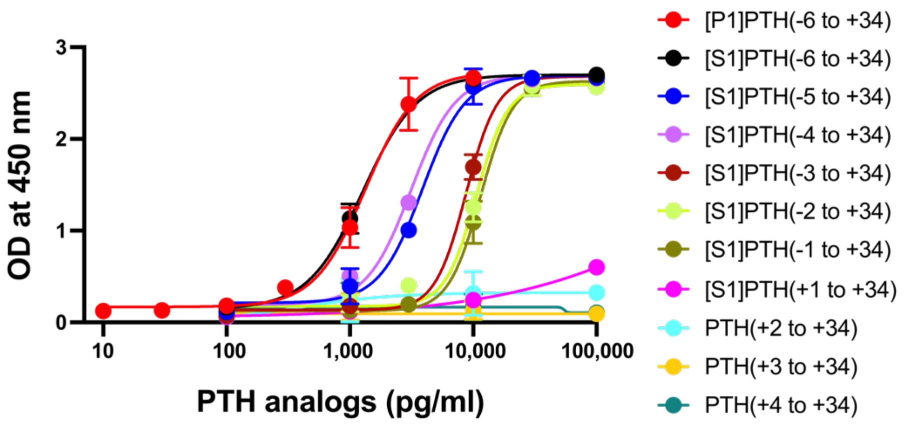

Fig. S3:

Defining the epitope of the antibody raised against [P1]PTH(-6 to +15) that was affinity purified with immobilized [P1]PTH(-6 to +3).

The anti-[P1]PTH(-6 to +3) antibody was coupled to biotin (anti-[P1]PTH(-6 to +3)^{Biotin} antibody) and used as capture antibody in combination with the detection anti-PTH(13-34) antibody provided in the intact PTH assay (Immutopics). Epitope of the affinity purified anti-[P1]PTH(-6 to +3)^{Biotin} antibody was partially defined by using [P1]PTH(-6 to +34) and [S1]PTH(-6 to +34), as well as peptides that were progressively shortened at the amino-terminus, including [S1]PTH(1-34), PTH(2-34), PTH(3-34), and PTH(4-34). While [P1]PTH(-6 to +34) (half maximum binding: 1,330 pg/ml, limit of detection: 37 pg/ml) and [S1]PTH(-6 to +34) were detected equally well in this assay, [S1]PTH(1-34) and PTH(2-34) showed only minimal cross-reactivity at very high concentrations, and PTH(3-34) and PTH(4-34) were not detected at all. These data indicate that the epitope recognized by the anti-[P1]PTH(-6 to +3)^{Biotin} antibody resides predominantly in the -6 to -4 region. (n = 2 to 4).

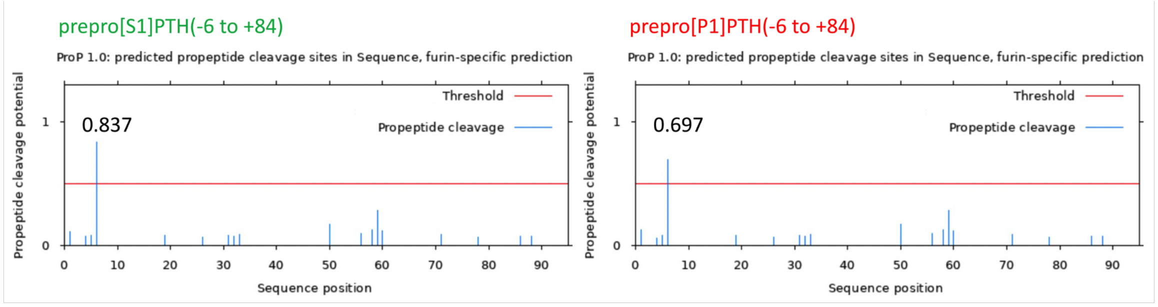

Fig. S4:

In silico prediction of cleavage of proPTH with either S1 or P1 by furin using ProP 1.0 (DTU Health Tech).

pro[S1]PTH(-6 to +84) and pro[P1]PTH(-6 to +84) are predicted to have the same cleavage site between residues -1 and +1 for the pro-peptide (blue vertical line). Sequence position 0 on the X-axis is amino acid -6 of proPTH.

Table S1:

Examples of variants in the precursors of peptide hormones that bind to class B G protein-coupled receptors.

Amino acid Variants of Unknown Significance (VUS) involving either in the first residues of mature peptides or VUS identified in gnomAD database for the putative furin cleavage sites preceding the secreted peptide. Gene name, chromosomal location based on assembly GRCh38/hg38, portions of the pro-sequences, as well as the first six amino acids of the mature peptides are shown. Allele frequencies of each variant affecting the normal amino acid (bold letters) are provided and it is indicated whether these are heterozygous (het) or homozygous (homo).

Name	Gene	Location hg38	Furin cleavage site	pro	Mature	VUS in secreted peptide	Allele frequency		pro-seq VUS	Allele frequency		Ref
Parathyroid Hormone	PTH	chr11:13492099-13496181	VKKR	KS**VK**KR	**SV**SEIQ...	p.Val33Leu	0.00000293	het	p.Val28Phe<br>p.Val28Ala<br>p.Lys29Arg<br>p.Lys29Asn	0.000003984<br>0.000002930<br>0.000003984<br>0.000003981	het	4
(Parathyroid Hormone 2) TIP39	PTH2	chr19:49422419-49423441	RPRR	TPR**PR**R	**SL**ALAD...	p.Ser62Gly<br>p.Leu63Arg<br>p.Leu63Pro	0.00001272<br>0.000005584<br>0.000005584	het<br>het<br>het	p.Pro59Ser<br>p.Pro59Thr<br>p.Arg60Leu	0.0009176<br>0.0005410<br>0.00004263	homo<br>homo<br>het	
Secretin	SCT	chr11:626309-627181	RARR	PPR**ARR**	**H**SDGTF...	p.His28Asn	0.00001597	het	p.Ala25_Arg26dup<br>p.Arg27Gln	0.000008228<br>0.000008093	het<br>het	
Vasoactive Intestinal Peptide	VIP	chr6:152750797-152759760	RNAR	VSRNAR	**H**SDAVF...	p.His125Gln	0.00003186	het				
Growth Hormone Releasing Hormone	GHRH	chr20:37251086-37261819	RMRR	LRM**RR**	Y**A**DAIF...	p.Ala33Val	0.000004003	het	p.Arg30Pro<br>p.Arg30Gln<br>p.Arg31Gln	0.0000040120<br>0.00001425<br>0.00001203	het<br>het<br>het	5
Gastric Inhibitory Polypeptide	GIP	chr17:48958554-48968596	RGPR	QPRGP**R**	**YA**EGTF...	p.Tyr52Phe<br>p.Ala53Pro<br>p.Ala53Thr<br>p.Ala53Val	0.000003976<br>0.000003977<br>0.000007953<br>0.00006364	het<br>het<br>het<br>homo	p.Arg51Thr	0.00001988	het	
Corticotropin Releasing Hormone	CRH	chr8:66176376-66178464	RERR	PERE**RR**	**S**E**E**PPI...	p.Ser154Tyr<br>p.Glu156Gly	0.000003986<br>0.000003983	het<br>het	p.Arg152Gly<br>p.Arg153Gly<br>p.Arg153Gln	0.00001597<br>0.000003991<br>0.000003989	het<br>het<br>het	6
Calcitonin	CALCA	chr11:14966669-14972286	RSKR	SPRSK**R**	C**G**NLST...	p.Gly86Ser	0.000007953	het	p.Arg84Trp<br>p.Arg84Gln<br>p.Arg84Pro	0.0001131<br>0.000003976<br>0.000003976	het<br>het<br>het	7

REFERENCES

1. J. Meienhofer, *et al.*, Solid phase synthesis without repetitive acidolysis. Preparation of leucyl-alanyl-glycyl-valine using 9-fluorenylmethyloxycarbonylamino acids. *Int J Pept Protein Res* **13**, 35–42 (1979).

2. D. S. King, C. G. Fields, G. B. Fields, A cleavage method which minimizes side reactions following Fmoc solid phase peptide synthesis. *Int J Pept Protein Res* **36**, 255–266 (1990).

3. M. Karas, F. Hillenkamp, Laser desorption ionization of proteins with molecular masses exceeding 10,000 daltons. *Anal Chem* **60**, 2299–2301 (1988).

4. Thakker R, Bringhurst F, Jüppner H. Regulation of calcium homeostasis and genetic disorders that affect calcium metabolism. In: DeGroot L, Jameson J, eds. *Endocrinology, Adult and Pediatric*. Vol 1. Philadelphia, PA: W.B. Saunders Company, 1063–1089 (2016).

5. Malagón MM, Vázquez–Martínez R, Martínez–Fuentes AJ, Gracia–Navarro F, Castaño JP. Growth Hormone-Releasing Hormone. In: Kastin AJ, eds. *Handbook of Biologically Active Peptides*. San Diego, California: Academic Press, 663–671 (2006).

6. I. Ahmed, *et al.*, Processing of Procorticotropin-Releasing Hormone (Pro-CRH): Molecular Forms of CRH in Normal and Preeclamptic Pregnancy1. *The Journal of Clinical Endocrinology & Metabolism* **85**, 755–764 (2000).

7. M. I. Hu and R. F. Gagel, Calcitonin Gene Family of Peptides, In: J. P. Bilezikian, L. G. Raisz and T. J. Martin.3rd eds. *Principles of Bone Biology*. Vol 1. San Diego, California: Academic Press, 813–836 (2008).
